# *ITGA2B*/*ITGB3*‐Related Macrothrombocytopenia Associated With Gain‐of‐Function Mutations in *ITGA2B* or *ITGB3* Genes

**DOI:** 10.1111/jcmm.70988

**Published:** 2026-01-08

**Authors:** Jiao Wu, Han Yan, Zijian Li, Yilin Zhu, Ruonan Shao, Honglei Xin, Tongyu Jia, Mengyu Ge, Lu Zhang, Suyu Jiang, Jianhua Mao, Jiansong Huang, Chao Fang, Xiaodong Xi, Xiaofeng Shi

**Affiliations:** ^1^ Department of Hematology The Second Affiliated Hospital of Nanjing Medical University Nanjing China; ^2^ Department of Hematology Chifeng Municipal Hospital Chifeng China; ^3^ Shanghai Institute of Hematology, State Key Laboratory of Medical Genomics, National Research Center for Translational Medicine at Shanghai, Collaborative Innovation Center of Hematology Ruijin Hospital, Shanghai Jiao Tong University School of Medicine Shanghai China; ^4^ Department of Hematology, Zhejiang Key Laboratory for Precision Diagnosis and Treatment of Hematological Malignancies, The First Affiliated Hospital Zhejiang University School of Medicine Hangzhou Zhejiang China; ^5^ Department of Pharmacology, School of Basic Medicine, Tongji Medical College and State Key Laboratory for Diagnosis and Treatment of Severe Zoonotic Infectious Diseases Huazhong University of Science and Technology Wuhan Hubei China; ^6^ The Key Laboratory for Drug Target Researches and Pharmacodynamic Evaluation of Hubei Province and Tongji‐Rongcheng Center for Biomedicine Huazhong University of Science and Technology Wuhan Hubei China

**Keywords:** gain‐of‐function mutation, Glanzmann thrombasthenia, integrin αIIbβ3, macrothrombocytopenia, platelet disorder

## Abstract

Glanzmann thrombasthenia (GT) is an inherited hemorrhagic disorder characterised by impaired platelet functions, manifested clinically as spontaneous bleeding. It is usually inherited in an autosomal recessive manner. Platelet dysfunction in patients with GT is caused by quantitative and/or qualitative deficiencies in αIIbβ3, which result from mutations in the genes encoding αIIbβ3. These genetic alterations lead to platelet dysfunction characterised by impaired fibrinogen binding capacity upon agonist stimulation, defective aggregation and spreading. While classical GT typically exhibits normal platelet counts and morphology, very rare mutations in *ITGA2B* (*encoding* αIIb) and/or *ITGB3* (*encoding* β3) cause macrothrombocytopenia or increased platelet anisotropy (heterogeneity of platelet size and morphology). This type of mutation mainly localises in the membrane‐proximal region of αIIbβ3 and is inherited in an autosomal dominant manner. This particular type of disorder is called *ITGA2B*/*ITGB3*‐related macrothrombocytopenia and has been considered a subset of congenital macrothrombocytopenia. Current research suggests that gain‐of‐function mutations in *ITGA2B* or *ITGB3* underlie the pathogenesis of most *ITGA2B*/*ITGB3*‐related macrothrombocytopenia and mechanistically distinguish it from classical GT. However, recent reports have documented non‐activating ITGB3 mutations that also cause macrothrombocytopenia, presenting a profound challenge to the mechanistic understanding of *ITGA2B/ITGB3*‐related macrothrombocytopenia. This review summarises the reported cases of gain‐of‐function mutations in *ITGA2B* and *ITGB3* associated with *ITGA2B*/*ITGB3*‐related macrothrombocytopenia hitherto and discusses the potential molecular pathways contributing to the unique phenotypes in *ITGA2B*/*ITGB3*‐related macrothrombocytopenia.

**Abbreviations**
CHO: Chinese hamster ovary cell
CRR: cysteine‐rich repeat region
CT: cytoplasmic tail
DTT: dithiothreitol
ECD: extracellular domain
EGF: cysteine‐rich growth factor
FAK: focal adhesion kinase
GT: Glanzmann thrombasthenia
IMC: inner membrane clasp
LIBS: ligand‐induced binding site
MIDAS: metal ion‐dependent adhesion site
MPV: mean platelet volume
OMC: outer membrane clasp
PAC‐1: a monoclonal antibody used to detect platelet activation status
PCR: polymerase chain reaction
PMA: phorbol‐12‐myristate‐13‐acetate
PPF: proplatelet formation
PSI: plexin‐semaphorin‐integrin
TMD: transmembrane domain
βTD: β‐tail domain

## 1 Introduction

Integrin αIIbβ3 is mainly expressed on the surface of platelets and megakaryocytes. αIIbβ3 is synthesised continuously during megakaryocytopoiesis (the production and maturation of megakaryocytes), spanning from the stage of haematopoietic stem cells to the stage of proplatelets [1]. Genetic defects of *ITGA2B* (*encoding* αIIb) or *ITGB3* (*encoding* β3) may damage the normal conformation and function of αIIbβ3, and ultimately impair megakaryocyte differentiation, proplatelet formation (PPF) and platelet functions. The *ITGA2B* and *ITGB3* are located on chromosome 17q21‐23. The *ITGA2B* gene segment is approximately 17 kilobases (kb) long with 30 exons, while the *ITGB3* gene segment is approximately 46 kb long with 15 exons. Therefore, in spite of the shorter gene segment on chromosome, *ITGA2B* contains more exons than *ITGB3*, and higher probabilities of missense mutations [2]. Large deletions are rare while nonsense and missense mutations are relatively common [3]. Mutations at splicing sites are often accompanied by mRNA instability or premature termination of polypeptide chain synthesis, resulting in truncated proteins and seriously affecting protein synthesis [4]. Because abnormal αIIb and β3 subunits are unable to form the normal αIIbβ3 complex, the uncomplexed αIIb or β3 subunit is rapidly degraded [2]. The abnormality of αIIb or β3 subunits can cause Glanzmann thrombasthenia (GT). GT can be classified into three types based on the levels of αIIbβ3: type I with < 5% of normal levels, type II with 10%–20% and type III (also called variant type). Type III has normal levels of αIIbβ3 but with functional defects, i.e., the glycoproteins are present but do not work correctly [5, 6]. For over two decades, cases of mutations in *ITGA2B* or *ITGB3* accompanied by spontaneous activation of αIIbβ3 and macrothrombocytopenia have been reported in clinical practice. These mutations predominantly cluster in the membrane‐proximal region of αIIbβ3 [7, 8, 9]. This region is critical for maintaining the bent inactive conformation of αIIbβ3 in resting platelets. Mutations in this region disturb this structural arrangement, forcing αIIbβ3 into a spontaneously activated state [10]. These mutations are defined as gain‐of‐function mutations, which lead to two major consequences: (1) constitutive activation of αIIbβ3, i.e., αIIbβ3 is spontaneously activated or easily activated in the absence of agonists; (2) impaired megakaryocytopoiesis, i.e., PPF and platelet release are impaired during megakaryocyte maturation due to defective cytoskeletal remodelling caused by aberrant integrin signalling. The controlled dynamics of αIIbβ3 conformation is the basis for ensuring its normal signalling and megakaryocytopoiesis.

## 2 Conformation of αIIbβ3 Underlie Its Function

αIIbβ3 is a heterodimeric calcium‐dependent glycoprotein receptor. Both α and β subunits are transmembrane glycoproteins and they form complexes through noncovalent interactions. Both subunits are composed of three distinct structural regions: extracellular domain (ECD), transmembrane domain (TMD) and short cytoplasmic tail (CT). The α subunit comprises an α heavy chain and a β light chain linked by a disulfide bond. The extracellular region consists of four domains: a seven‐bladed β‐propeller, a thigh domain and two calf domains [11, 12]. The β subunit is a cysteine‐rich single‐chain protein. The extracellular region contains seven complex domains: a βI‐like/βA domain inserted within a hybrid structural domain, a plexin‐semaphorin‐integrin (PSI) domain, four cysteine‐rich epidermal growth factor (EGF)‐like modules and a beta‐tail domain (βTD). The βA domain of the β subunit contains the metal ion‐dependent adhesion site (MIDAS) that directly coordinates ligand binding, forms the core of the ligand‐binding pocket in conjunction with the β‐propeller of the α subunit, and undergoes conformational activation to enable high‐affinity recognition of Arg‐Gly‐Asp (RGD) motifs in fibrinogen and other ligands [11, 12, 13, 14].

There are three different states of αIIbβ3 depending on their conformations: a bent‐closed (inactive) form, an extended‐closed (intermediate) form, and an extended‐open (active) form [14]. In resting platelets, the bent‐closed conformation of αIIbβ3 is stabilised by structural constraints imposed by its transmembrane and cytoplasmic domains, which are called the “outer membrane clasp” (OMC) and “inner membrane clasp” (IMC), respectively. The IMC contains two forces formed between the αIIb G^991^F^992^F^993^KR^995^ (Gly^991^Phe^992^Phe^993^LysArg^995^) motif and β3 HD^723^R(R/K) E [HisAsp^723^Arg (Arg/Lys) Glu] motif: (1) stacking forces formed by the two aromatic rings Phe^992^ and Phe^993^ on αIIb and (2) electrostatic interactions formed between the Arg^995^ guanidino group on αIIb and the Asp^723^ carboxyl group on β3. The latter is called the αIIbArg^995^/β3Asp^723^ salt bridge. OMC is a kind of stacking force formed by the mutually oriented juxtaposition of the motif sequence Gly^972^Gly^976^Leu^980^ and the motif sequence Val^700^Ile^704^Gly^708^ of the respective α helix of the transmembrane domain of αIIb and β3 subunits. The activation of αIIbβ3 requires disruption of the αIIb‐Arg^995^/β3‐Asp^723^ salt bridge within the IMC. This conformational switch enables the transition from a bent‐closed (inactive) to an extended‐open (active) state, facilitating ligand binding and platelet aggregation [14, 15, 16]. The inside‐out signalling pathway involves the dissociation of the transmembrane portion of the integrin heterodimer, rearrangement of the ligand‐binding domains and increase of integrin binding affinity that ultimately allows for the binding to a variety of different adhesion proteins [11, 17, 18].

## 3 Integrin αIIbβ3‐Regulated Cytoskeletal Reorganisation Governs Thrombopoiesis

In the bone marrow niche, megakaryocytes undergo multiple rounds of mitotic divisions from 2 N to 128 N polyploid megakaryocytes, accompanied by cytoplasmic expansion, organelle biogenesis and surface membrane amplification. Driven by gradient concentrations of chemokines such as stromal cell‐derived factor 1 and fibroblast growth factor, megakaryocytes with pseudopods move into the perivascular space through a process dependent on myosin‐driven actin reorganisation [19]. After entering the vascular niche, mature megakaryocytes form bulges called proplatelets, which extend through the sinusoidal wall of the blood vessel and are shed off as disc‐shaped preplatelets under the shear force of blood flow. Preplatelets can be reversely transformed into barbell‐like platelets, which are characterised as an “8” structure with a twisted tubulin skeleton around the center. Preplatelets eventually split to form two mature platelets through a process that is accelerated by the shear force of blood flow [20]. During the process of proplatelet formation, cytoskeleton proteins, including tubulin, actin and motor proteins (including myosin, kinesin and dynein), play essential roles. The main driving force of the elongation of the proplatelet shaft is microtubule sliding driven by dynein [21]. β1‐tubulin is expressed exclusively in megakaryocytes and platelets and is the major tubulin involved in PPF. Mutations in *TUBB1* disrupt PPF, causing macrothrombocytopenia through impaired platelet maturation [22, 23, 24, 25]. During the formation of proplatelets, organelles and particles are arranged along the bipolar tubulin of the long axis of the proplatelets, and the movement and mutual sliding of tubulin promote the transport of particles and organelles to the tips of the proplatelets. These particles and organelles ultimately participate in the formation of the complete structure of functional mature platelets [26]. Besides the tubulin system, actin primarily facilitates the proplatelet branching, therefore amplifying the number of terminal tips. Defects or abnormalities of proteins associated with actin dynamics can result in thrombocytopenia and/or abnormal platelet morphology [25, 27, 28, 29].

Whether αIIbβ3‐mediated signalling is involved in the production of platelets by megakaryocytes is still under debate. The binding of fibrinogen to αIIbβ3 has been shown to be essential for the formation of proplatelets in mouse megakaryocytes in a manner independent of the inside‐out signalling pathway of αIIbβ3 [30]. It was also previously reported in humans that the interaction between fibrinogen and αIIbβ3 was not essential for proplatelet formation [31]. Although constitutive activation of αIIbβ3‐mediated outside‐in signalling negatively influences proplatelet formation and leads to macrothombocytopenia [32], GT is not associated with thrombocytopenia but only quantitative and/or qualitative abnormalities of αIIbβ3 [33, 34].

## 4 Congenital Macrothrombocytopenia Caused by Mutations of *ITGA2B* and *ITGB3*

**FIGURE 1:**
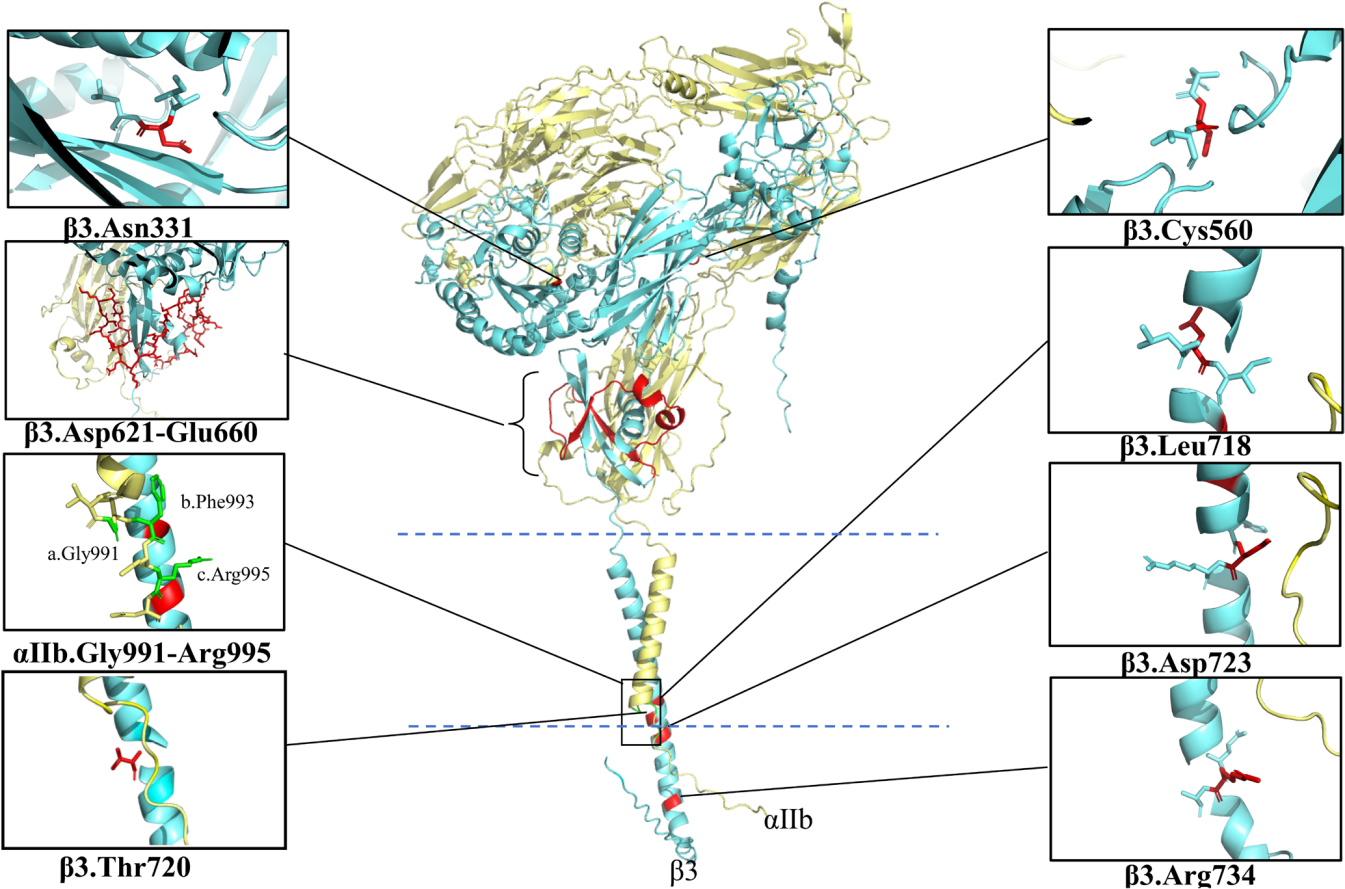
Schematic diagram of the gain‐of‐function mutation sites in *ITGA2B* and *ITGB3*, including missense mutations and deletions. β3Arg^734^‐to‐Cys has been shown to be a non‐activating mutation in the latest study. The yellow helical structure represents αIIb, and the blue represents β3. The 3D structural modelling of the protein was generated by AlphaFold Server (https://alphafoldserver.com).

Congenital macrothrombocytopenia is observed in individuals with *MYH9* disorders, heterozygous and homozygous Bernard‐Soulier syndrome, type 2B von Willebrand disease, *TUBB1* mutations, *ACTIN1* mutations, etc. [2, 25, 27]. The pathogenesis of nearly half of congenital macrothrombocytopenia cases is still unknown, and treatment strategies for this condition still need further investigation. We have summarised the clinical characteristics of cases reported to date. In these patients, gain‐of‐function mutations in the *ITGB3* or *ITGA2B* genes lead to spontaneous activation of αIIbβ3 (summarised in Table 1 and Figure 1). These mutations primarily localise at the membrane‐proximal regions and induce different degrees of platelet dysfunction. This disorder is designated as GLTS to distinguish it from classical GT.

**TABLE 1:** Mutations in ITGA2B and ITGB3 with evidence of αIIbβ3 spontaneous activation.

Report time and reference	Mutation position			Platelet count (×10^9^ /L)	Platelet size and morphology	Surface expression of αIIbβ3 (% of normal)	Evidence of spontaneous activation
	Mutation gene	Variant protein	Protein region				
1992 [35, 36]	ITGA2B	Arg995‐to‐Gln	Salt‐bridge	100–160	platelet anisotropy and giant platelet (volume 10.3 μm^3^ versus 8.6 ± 1 μm^3^)	15–20	Increased AI (transfected CHO cell)
		(Arg1026‐to‐Gln)^a^					
2011 [37]	ITGA2B	Arg995‐to‐Trp	Salt‐bridge	65–122	large platelet (diameter 3.2–3.9 μm versus 2.5 μm)	50–70	Spontaneous combination with PAC‐1 (patient's platelet) and spontaneous FAK phosphorylation (transfected 293T cell)
		(Arg1026‐to‐Trp)^a^					
2008 [38]	ITGB3	Asp723‐to‐His	Salt‐bridge	80	large platelet (MPV 17 fL versus 12–13 fL)	Decreased	Spontaneous combination with PAC‐1 (patient's platelet and transfected CHO cell)^b^
		(Asp749‐to‐His)					
2013 [39]	ITGA2B	Gly991‐to‐Cys	CT	22–102	large platelet (diameter 3.4 ± 0.8 μm versus 2.5 ± 0.3 μm)	3–11	Spontaneous combination with PAC‐1 and spontaneous FAK phosphorylation (transfected 293T cell)^b^
		(Gly1022‐to‐Cys)^a^					
2013 [39]	ITGA2B	Phe993del	CT	59–111	large platelet (diameter 3.4 ± 1.2 μm versus 2.5 ± 0.3 μm)	74–78	Spontaneous combination with PAC‐1 and spontaneous FAK phosphorylation (transfected 293T cell)^b^
		(Phe1024del)^a^					
2020 [8]	ITGB3	Arg734‐to‐Cys	CT	115	Large platelet (MPV 13 fL versus 7–11 fL)	77	Spontaneous combination with PAC‐1 (patient's platelet)^b^
		(Arg760‐to‐Cys)^a^					
2010 [40]	ITGB3	Leu718‐to‐Pro	TMD	127	Platelet anisotropy	47	Spontaneous combination with PAC‐1 (transfected CHO cell)^b^
		(Leu744‐to‐Pro)^a^					
2013 [41]	ITGB3	Leu718‐to‐Pro	TMD	49–72	Normal	43–75	Spontaneous combination with PAC‐1 (patient's platelet and transfected CHO cell)^b^
		(Leu744‐to‐Pro)^a^					
2018 [42]	ITGB3	Thr720del	TMD	58–86	Large platelet (MVP 12.8–14.5 fL)	40	Spontaneous combination with PAC‐1 (patient's platelet) and spontaneous FAK phosphorylation (transfected CHO cell and 293T cell)^b^
		(Thr746del)^a^					
2001 [43]	ITGB3	Cys560‐to‐Arg	EMD	100–150	Platelet anisotropy	20	Spontaneous combination with PAC‐1 (patient's platelet and transfected CHO cell)^b^
		(Cys586‐to‐Arg)^a^					
2009 [44]	ITGB3	Asp621‐Glu660 deletion	EMD	26–79	large platelet (MVP 14.2 fL)	Decreased	Spontaneous combination with PAC‐1 (patient's platelet)^b^
		(Asp647‐Glu686 deletion)^a^					
2013 [39]	ITGB3	Asp621‐Glu660 deletion	EMD	29–113	large platelet (diameter 5.1 ± 1.0 μm versus 2.5 ± 0.3 μm)	67	Spontaneous combination with PAC‐1 (transfected 293T cell)^b^
		(Asp647‐Glu686 deletion)^a^					
2018 [45]	ITGB3	Asn331‐to‐Ser	EMD	64–90	large platelet (MVP 13 fL versus 8–12 fL)	Decreased	Spontaneous combination with PAC‐1 (patient's platelet and transfected CHO cell)^b^
		(Asn357‐to‐Ser)^a^					

*Note:* β3Arg^734^‐to‐Cys has been shown to be a non‐activating mutation in the latest study.

Abbreviations: AI, Activation index (relative PAC‐1 binding in resting platelets compared with maximal PAC‐1 binding in platelets stimulated with PMA); CT, cytoplasmic tail; ECD, extracellular domain; TMD, transmembrane domain.

^a^ The residue numbering in parentheses reflects positions calculated inclusive of their respective signal peptide sequences (αIIb subunit: 31 residues; β3 subunit: 26 residues).

^b^ Spontaneous binding to PAC‐1 refers to binding to PAC‐1 in the absence of stimulants.

### 4.1 Mutations in the Cytoplasmic Tail of αIIbβ3

#### 4.1.1 Mutations in the Region of Salt Bridges

Hardisty et al. [35] reported for the first time a case of a compound heterozygous patient, with a paternal αIIbArg^995^‐to‐Gln mutation and a maternal splicing defect mutation (c.1440‐13_1440‐1del) [7, 36]. The patient presented with mucocutaneous bleeding, severely reduced αIIbβ3 surface expression on resting platelets (15%–20%), but the level of surface αIIbβ3 became essentially normal after thrombin stimulation, indicating intact internal pool mobilisation. The platelet count was normal but with heterogeneity in size, including giant forms. Platelet aggregation induced by adenosine diphosphate (ADP), collagen or thrombin was significantly reduced [35]. The CHO cell line stably expressing mutant αIIbArg^995^‐to‐Gln and wild‐type β3 showed no spontaneous integrin activation tested by PAC‐1 binding experiments, but normal activation in the presence of the activating antibody anti‐LIBS6. However, enhanced activation index (relative PAC‐1 binding in resting platelets compared with maximal PAC‐1 binding in platelets stimulated with PMA) of mutant αIIbβ3 was detected (23 versus 8), indicating that αIIbArg^995^‐to‐Glnβ3 was more easily activatable, but its degree of activation was weaker than that of other artificial mutations (such as Arg^995^‐to‐Ala) [16, 36].

In 2011, Kunishima et al. [37] reported a novel conserved heterozygous αIIb Arg^995^‐to‐Trp mutation in 4 unrelated pedigrees in Japan. The patients presented with mild thrombocytopenia (platelet count 65–122 × 10^9^/L), increased platelet volume (3.2–3.9 μm versus 2.5 μm in diameter), and 50% to 70% of normal levels of αIIbβ3 expression on the platelet surface. The αIIbArg^995^‐to‐Trp mutation is the most common mutation type of *ITGA2B*/*ITGB3*‐related macrothrombocytopenia in Japan. Unlike Arg^995^‐to‐Gln, this mutation induced spontaneous partial αIIbβ3 activation, characterised by PAC‐1/fibrinogen binding without degranulation, along with spontaneous phosphorylation of focal adhesion kinase (FAK). It also disrupted megakaryopoiesis, with evidence from transfected cell models showing a reduced number of proplatelet tips and increased platelet volume. These findings represent the first direct linkage of the mutation to macrothrombocytopenia. They established the first murine model corresponding to the human αIIbArg^995^‐to‐Trp mutation by knocking in the αIIbArg^990^‐to‐Trp mutation in mice. This model demonstrated that spontaneously activated αIIbβ3 disrupts proplatelet formation, leading to macrothrombocytopenia. Megakaryocyte development (including quantity, ploidy and morphology) remains unaffected. Platelet dysfunction and shortened platelet lifespan coexist, with the latter exacerbating thrombocytopenia. Persistent activation of αIIbβ3 likely triggers enhanced internalisation and lysosomal degradation, resulting in downregulated αIIbβ3 expression in platelets surface [38]. Although both mutations occurred at the same residue (Arg^995^), their distinct amino acid substitutions led to different biological outcomes. The Arg^995^‐to‐Gln substitution primarily reduced αIIbβ3 surface expression on platelets while rendering the αIIbβ3 receptor with an increased ability to be activated. In contrast, the Arg^995^‐to‐Trp mutation disrupted platelet production by megakaryocytes and induced spontaneous αIIbβ3 activation without triggering platelet activation. These differences were likely attributed to the distinct charge properties and steric effects of the substituted amino acids.

Ghevaert et al. [46] identified a heterozygous β3Asp^723^‐to‐His mutation in a family with an autosomal dominant disorder. This heterozygous mutation, disrupting the same αIIbArg^995^/β3Asp^723^ salt bridge, also caused macrothrombocytopenia. It led to spontaneous partial αIIbβ3 activation and, critically, induced aberrant proplatelet formation in megakaryocytes which derived from the proband's CD34+ stem cells. Notably, it was proposed for the first time that this defect was driven by αIIbβ3‐mediated RhoA/ROCK inhibition and microtubule reorganisation, directly connecting constitutive activation of αIIbβ3 to cytoskeletal dysregulation underlying the platelet production defect. They also observed no early defects in megakaryocyte maturation, no difference in ploidy distribution (2 N–32 N) from the control, but abnormal proplatelets with enlarged tips and reduced branches, indicating abnormalities arising during the terminal maturation stage of megakaryopoiesis [47]. Enlarged α‐granules were also noted. They speculated that it may originate from abnormal secretory pathways or particle fusion [39].

#### 4.1.2 Mutations Near the Region of Salt Bridges

The G^991^FFKR^995^ of αIIb and the L^717^LITIHD^723^ of β3 are conserved sequences in the membrane‐proximal region, participating in the formation of salt bridges and maintaining the stability of the transmembrane helix structure [16]. Kashiwagi et al. [48] reported two mutations in the GFFKR region of αIIb, Gly^991^‐to‐Cys and Phe^993^del. The first case was a compound heterozygous mutation Gly^991^‐to‐Cys and Arg^422^* (Arg^422^ mutated to stop codon) of *ITGA2B*, resulting in only 3% to 11% of αIIbβ3 surface expression and 60% retention within the cell, and a severe GT‐like phenotype with macrothrombocytopenia. The second case was a heterozygous mutation Phe^993^del of *ITGA2B*, leading to a slight decrease in αIIbβ3 expression (74%–78%) and an increase in platelet volume. 293T cells expressing either type of mutated αIIbβ3 showed spontaneous activation of αIIbβ3 (increased PAC‐1 binding) and sustained phosphorylation of FAK. The mutations disrupted the stable resting conformation of αIIbβ3, leaving the integrin in a state with sustained spontaneous activation, therefore interfering with megakaryocyte maturation and platelet release. Certain segments within the conserved domains G^991^FFKR^995^ of αIIb and L^717^LITIHD^723^ of β3 are embedded in the platelet membrane, therefore contributing to the stabilisation of the relative stereoscopic distribution of the transmembrane helices. The arrangement of these predominantly hydrophobic residues dictates the angle of inclination between the transmembrane helices, ultimately influencing the angle of alignment by which αIIb and β3 face each other. Furthermore, Phe^992^ and Phe^993^ are involved in the formation of IMC and work together to maintain the bent conformation of αIIbβ3 [40, 49].

In 2020, two pedigrees with β3 Arg^734^‐to‐Cys (Arg^760^‐to‐Cys in the original article where 26 leading amino acids were included) heterozygous mutations were reported [8]. This mutation is located in the distal cytoplasmic tail of β3. In the probands, platelet count was comparable to normal levels (115 × 10^9^/L), mean platelet volume (MPV) slightly increased (13 fL), and αIIbβ3 expression was close to the lower limit of the normal range (77%). Platelet aggregations were slightly reduced in response to most physiological agonists but normal under the stimulation of ADP. In the resting state, the patient's platelets had a higher level of PAC‐1 binding but normal fibrinogen binding, indicating that this activation was “ineffective”. However, recent studies utilising mouse models have demonstrated that β3Arg^734^‐to‐Cys mutations caused macrothrombocytopenia without inducing spontaneous activation of αIIbβ3. This provided the first evidence that a non‐activating *ITGB3* mutation could lead to macrothrombocytopenia. Crucially, these studies directly proved that the mutation disrupted the αIIbβ3/RhoA/cytoskeletal signalling axis, thereby impairing proplatelet formation in megakaryocytes. While the involvement of the αIIbβ3/RhoA/cytoskeletal signalling axis in proplatelet formation by megakaryocytes had been established previously, this study challenged the conventional view that spontaneous integrin activation was a prerequisite for macrothrombocytopenia. The shortening of platelet lifespan aggravates thrombocytopenia [41].

### 4.2 Mutations in the Transmembrane Region of αIIbβ3

Jayo et al. [50] reported a 43‐year‐old female from Spain, carrying a heterozygous Leu ^718^‐to‐Pro mutation in the transmembrane region of β3. This individual had a history of recurrent severe cutaneous and mucosal bleeding since childhood, accompanied by a normal platelet count (mean platelet count 127 × 10^9^/L) but platelet anisocytosis. Platelet aggregation showed diminished responses (10%–20% compared to controls) when stimulated with ADP, epinephrine, collagen or arachidonic acid, but a normal response when stimulated with ristocetin. Spreading on fibrinogen was normal. The CHO cells transfected with the β3Leu^718^‐to‐Pro mutation and αIIb spontaneously bound to PAC‐1 and fibrinogen, but demonstrated significantly reduced activation response when treated with dithiothreitol (DTT), which promotes conformational change by reducing disulfide bonds on αIIb and β3. It is possible that the αIIbβ3Leu^718^‐to‐Pro was already in a state of maximum or near‐maximum activation and DTT could not induce further conformational changes. It is also possible that the abnormal aggregation of αIIbβ3Leu^718^‐to‐Pro limited conformational flexibility; therefore, making the integrin mutant less susceptible to DTT reduction.

Kobayashi et al. [42] identified six individuals across a four‐generation pedigree carrying the same β3Leu^718^‐to‐Pro mutation, accompanied by mild to moderate thrombocytopenia but normal platelet size. Flow cytometry revealed that the surface expression of αIIbβ3 on patients' platelets was reduced to 43%–75% of normal control levels, while western blot analysis detected both a normal β3 band and a lower molecular‐weight band, suggesting potential abnormal cleavage or degradation of the mutant protein. Resting platelets from patients exhibited increased binding to PAC‐1 and fibrinogen, with impaired platelet spreading and aggregation, consistent with the findings previously reported by Jayo et al. In CHO cells transfected with the β3Leu^718^‐to‐Pro mutant, αIIbβ3 was partially activated and promoted the generation of abnormal proplatelet‐like protrusions by downregulating RhoA activity. These results further support the gain‐of‐function mechanism underlying thrombocytopenia via RhoA/Rock inhibition. However, deletion of the same locus (Leu^718^) by in‐frame heterozygous 3‐bp deletion (c.2230_2232delCTC) caused moderate macrothrombocytopenia in an autosomal dominant manner. The patient's platelets showed decreased but not spontaneous activation of αIIbβ3 and enlarged α‐granules [43].

Additionally, a heterozygous mutation Thr^720^del by in‐frame heterozygous 3‐bp deletion (*ITGB3*, c.2236_2238delACC) in the cytoplasmic transmembrane region of β3 was identified. The mutation disrupted the structural stability of αIIbβ3 and triggered its spontaneous activation. This mutation was first reported in a Japanese family presenting with mild thrombocytopenia (platelet count 58–86 × 10^9^/L), increased mean platelet volume (MPV 12.8–14.5 fL), and markedly reduced platelet aggregation in response to ADP and collagen. While surface expression of αIIbβ3 on patient's platelets was reduced to 40% of normal level, total cellular αIIbβ3 expression remained normal. Notably, resting mutant platelets exhibited constitutive αIIbβ3 activation, manifested by spontaneous binding to PAC‐1 and an increase in activation index by 14%. Expression of the αIIbβ3Thr^720^del mutant in 293 T and CHO cells resulted in spontaneous FAK phosphorylation and aberrant cellular morphology, characterised by rhomboid shape transformation and cytoplasmic protrusions [51]. Two other missense mutations (Thr^720^ to Pro and His^722^ to Pro) occurring in the TIH motif of β3 exhibited phenotypic features of macrothrombocytopenia. However, no evidence of spontaneous integrin activation was observed [8].

Recently, Lee et al. [9] reported an *ITGB3* mutation Leu^705^‐to‐Arg (Leu^731^‐to‐Arg in the original article where the leading 26 amino acids were included) in a patient who presented with no significant bleeding manifestations but thrombocytopenia (51 × 10^9^/L), increased MPV (11 fL), and platelet anisocytosis on blood smear. Platelet aggregation responses to ADP, collagen, arachidonic acid and epinephrine were markedly reduced. Although functional assays to evaluate spontaneous activation of integrin αIIbβ3 were not conducted, the current evidence supports the diagnosis of *ITGA2B*/*ITGB3*‐related macrothrombocytopenia.

### 4.3 Mutations in the Region of the Extracellular Domain of β3

Ruiz et al. [44] identified for the first time a mutation in the third cysteine‐rich repeat region (CRR) of β3 that resulted in sustained activation of integrin. The proband had mild cutaneous mucosal bleeding, a nearly normal platelet count (100–150 × 10^9^/L), but increased platelet anisotropy. Platelet aggregation was severely impaired (no response to ADP, collagen, etc.), the clot retraction of whole blood and plasma was significantly reduced and the expression of αIIbβ3 on the surface decreased by 80%. Genomic sequencing identified a homozygous mutation in exon 10 of *ITGB3* (g1776T>C), resulting in the substitution of Cys^560^ to arginine (Cys^560^‐to‐Arg). The mutation induced a constitutively active conformation of αIIbβ3, as evidenced by two features: (1) spontaneous ligand binding as demonstrated by enhanced binding of PAC‐1 and fibrinogen in the absence of agonist stimulation; (2) constitutive exposure of ligand‐induced binding sites (LIBS epitopes) as revealed by immunostaining of the epitopes typically unmasked only during integrin activation. Using electron microscopy, fibrinogen was observed to bind to the surface of unstimulated platelets, while ultrastructural analysis confirmed the absence of morphological signatures of activated platelets. CHO cells expressing the αIIbβ3Cys^560^‐to‐Arg mutant integrin also exhibited spontaneous PAC‐1 binding, accompanied by significantly enhanced adhesion and spreading on fibrinogen. It has been demonstrated that the cysteines in the CRR of β3 are not all paired in the format of disulfide bonds. There are 2–3 free cysteines in the resting state and 4–5 free cysteines in the activated state, which indicates that the activation of αIIbβ3 is accompanied by the rearrangement of disulfide bonds in the CRR of β3 [45]. The mutation resulted in the rearrangement of disulfide bonds, mimicking the conformational switch during αIIbβ3 activation.

In 2009 Gresele et al. [52] described two Italian pedigrees who presented with moderate to severe cutaneous and mucosal bleeding, reduced platelet count (26–79 × 10^9^/L), increased MVP (14.2 fL) and an increased proportion of large and giant platelets (16%). Under electron microscope, increased platelet volume and increased number of α‐granules and δ‐granules were observed. Platelet aggregation was significantly impaired in response to ADP and epinephrine, slightly decreased in response to collagen, completely absent following TXA2 induction, but normal when stimulated with ristocetin. The collagen/ADP closure time evaluated by PFA‐100 was markedly prolonged. At high shear rates, platelet adhesion was impaired, but clot retraction was normal. The expression of αIIbβ3 on the surface of platelets was decreased under both resting state and when stimulated by TRAP‐6, but the expression of αIIbβ3 in the intracellular pool of platelets was normal. Fibrinogen and PAC‐1 binding to resting platelets was slightly enhanced. Western blot analysis of the proband's platelet lysate revealed a normal β3 band and a lower molecular‐weight band. Genome‐wide linkage analysis and sequencing identified a G>C conversion (C.2134+1G>C) within the splicing site of intron 13 of the *ITGB3* gene, resulting in a 120 bp deletion of exon 13. This mutation led to the absence of 40 amino acid residues (Asp^621^‐Glu^660^) in the membrane‐proximal β‐tail domain of β3. These phenotypes and the autosomal dominant inheritance were similar to the spontaneous activation of αIIbβ3 described above, although there was no direct evidence of spontaneous activation of αIIbβ3 provided in the study.

Subsequently, a different mutation at the same site was found in a Japanese family, namely C.2134+1G>A in the *ITGB3* gene. It also resulted in the deletion of 40 amino acid residues (Asp^621^‐Glu^660^) in the membrane‐proximal β‐tail domain of β3 [48]. The expression of αIIbβ3 on the surface of platelets was 67% of the control, and western blot analysis indicated that the patient's platelets contained normal and low molecular‐weight β3. The evidence for spontaneous activation of αIIbβ3, such as increased PAC‐1 binding and sustained FAK phosphorylation, was detected in 293 T cells transfected with the αIIbβ3 integrin mutant with Asp^621^‐Glu^660^ deletion.

In 2018, Bury et al. [53] reported a compound heterozygous of two *ITGB3* variants: c.2356C>T, leading to Arg^760^‐to‐Trp (Arg^786^‐to‐Trp in the original article where the leading 26 amino acids were included) in the β3 cytoplasmic tail, and c. 992A>G, leading to Asn^305^‐to‐Ser (Asn^331^‐to‐Ser in the original article where the leading 26 amino acids were included) in the βI‐like domain of the β3 globular head. The mutation of Arg^760^‐to‐Trp was found in the mother and brother, while the mutation of Asn^305^‐to‐Ser was found in the father and sister. The proband had a history of lifelong mild bleeding with a mildly reduced platelet count (64–90 × 10^9^/L) and increased MPV (13 fL). Electron microscopy confirmed increased platelet volume and increased anisotropy. The expression of αIIbβ3 on the platelet surface was decreased. Platelet aggregation was impaired under various agonists. Compared to the control group, platelet spreading was enhanced in the early stage (15 min) but reduced in the late stage (60 min). In platelet suspension, the phosphorylation of FAK was enhanced. When examined in CHO cells, the expression of αIIbβ3Asn^305^‐to‐Ser alone or combined with αIIbβ3Arg^760^‐to‐Trp induced enhanced internalisation, constitutive activation of αIIbβ3 and cytoskeletal abnormalities. In contrast, αIIbβ3Arg^760^‐to‐Trp expression alone only affected outside‐in signalling without exhibiting a dominant effect. Megakaryocytes derived from CD34+ cells with the β3Asn^305^‐to‐Ser mutation showed normal maturation but abnormal spreading on fibrinogen, reduced number but increased volume of proplatelet tips, and disorganised cytoskeleton under electron microscopy. Notably, classical GT is inherited in an autosomal recessive manner, which requires biallelic mutation, while this gain‐of‐function mutation exerts a dominant effect, resulting in an autosomal dominant GLTS phenotype.

## 5 Discussion

The αIIbArg^995^/β3Asp^723^ salt bridge is a critical structure for maintaining the resting conformation of αIIbβ3. While most mutations causing spontaneous activation of αIIbβ3 are localised near this region, some do occur in membrane‐proximal or extracellular domains. Mutations that disrupt the resting conformation of αIIbβ3 thus lead to spontaneous activation or a primed state are defined as gain‐of‐function mutations. These mutations exhibit dominant effects over wild‐type or loss‐of‐function variants. Functional studies of αIIbβ3 bearing various mutation sites are primarily conducted using CHO or 293 T cell models, in which integrin activation was assessed easily through PAC‐1 binding assays or FAK phosphorylation analysis. PAC‐1 is a monoclonal antibody that specifically binds to the exposed epitope of αIIbβ3 in the activated conformation and can serve as a molecular probe for the activated state of αIIbβ3. Spontaneous PAC‐1 binding or elevated activation index provides direct evidence of spontaneous activation of αIIbβ3. FAK phosphorylation primarily responds to αIIbβ3 outside‐in signalling and has been demonstrated to negatively regulate megakaryocyte maturation by inhibiting TPO signalling, thereby reducing platelet production [54, 55]. Early studies using CHO cells demonstrated that partial activation mutations in αIIbβ3 downregulate RhoA activity and induce microtubule‐dependent proplatelet‐like extensions [42, 47]. Activation of RhoA is critical for the final stage of platelet production. Under physiological conditions, downregulation of active RhoA is a prerequisite for microtubule polymerisation during PPF or neurite outgrowth [56, 57, 58]. Gain‐of‐function mutations in αIIbβ3 downregulate RhoA activity, relieving its inhibitory effect on PPF. This leads megakaryocytes to form precocious proplatelets, resulting in the premature and/or ectopic release of platelets within the bone marrow niche. Platelets prematurely released within the bone marrow niche may fail to enter the circulation effectively or undergo premature clearance, resulting in reduced platelet counts in the peripheral blood [47, 56]. Furthermore, spontaneous activation of αIIbβ3 leads to excessive actin polymerisation with impaired depolymerization and aberrant microtubule reorganisation. This ultimately results in megakaryocytes producing proplatelets with fewer tips, increased size and asymmetry, while also impairing platelet aggregation and spreading functions [59, 60]. However, recent studies using knock‐in mice demonstrate that non‐activating αIIbβ3 mutations can also cause macrothrombocytopenia by disrupting the RhoA/cytoskeletal axis, providing the first evidence that constitutive αIIbβ3 activation is not an essential prerequisite for this disorder. Crucially, it is now established that both gain‐of‐function mutations and non‐activating mutations induce macrothrombocytopenia through suppression of the αIIbβ3/RhoA/cytoskeletal signalling axis [41]. Downregulation of RhoA activity represents the common core mechanism, with cytoskeletal abnormalities being the most direct pathological consequence. Although the two types of αIIbβ3 mutations downregulate RhoA through different molecular mechanisms, targeted modulation of the RhoA/ROCK pathway may represent a therapeutic strategy for *ITGA2B/ITGB3*‐related macrothrombocytopenia.

## 6 Prospect

Although the central role of the RhoA/cytoskeletal axis in αIIbβ3 mutation‐induced macrothrombocytopenia has been initially established, future studies still require deeper exploration. The specific molecular pathways through which distinct mutation types dysregulate RhoA activity remain poorly defined, particularly regarding the dynamic interactions between intracellular adaptor proteins and mutant integrins. Additionally, further exploration is required to elucidate the spatiotemporal regulation of RhoA effectors during megakaryocyte spatial polarisation and platelet release. This should integrate high‐resolution live‐cell imaging to track the coordinated reorganisation of cytoskeletal components, including microtubules and actin filaments. Subsequent in vivo validation using mutant mice will assess whether targeted modulation of the RhoA/ROCK pathway can rescue mutation‐induced platelet morphological and numerical abnormalities. Ultimately, these efforts will enable precision therapeutic targeting based on mutational profiles, thereby improving clinical management of *ITGA2B/ITGB3*‐related macrothrombocytopenia.

## Author Contributions

**Jiao Wu:** writing – original draft (leading), visualisation (equal), conceptualization (equal). **Han Yan:** writing – original draft (supporting). **Zijian Li:** visualisation (equal), writing – review and editing (equal). **Yilin Zhu:** writing – original draft (supporting). **Ruonan Shao:** writing – review and editing (equal). **Honglei Xin:** writing – original draft (supporting). **Tongyu Jia:** writing – review and editing (equal). **Mengyu Ge:** writing – review and editing (equal). **Lu Zhang:** writing – review and editing (equal). **Suyu Jiang:** writing – review and editing (equal). **Jianhua Mao:** writing – review and editing (equal). **Jiansong Huang:** writing – review and editing (equal). **Chao Fang:** writing – review and editing (equal), supervision (equal). **Xiaodong Xi:** writing – review and editing (equal), supervision (equal). **Xiaofeng Shi:** writing – review and editing (equal), conceptualization (equal), supervision (equal).

## Funding

This article is supported by the National Natural Science Foundation of China (81700130, 82370134, 81970112, 82070118, 92169114, 81802932), the Science and Technology Commission of Shanghai Municipality (23S11900400, 23ZR1439900), the Natural Science Fund for Distinguished Young Scholars of Hubei Province (2022CFA054), NHC Key Laboratory of Thrombosis and Hamostasis, the First Affiliated Hospital of Soochow University (KJS2419) and the Undergraduate Teaching and Research Project (2024130) and Graduate Teaching and Research Project (2025YB015) of Huazhong University of Science and Technology, the National Natural Science Foundation of China (82530115).

## Ethics Statement

This review article did not involve direct human/animal subjects research or primary data collection. All referenced studies were ethically conducted in accordance with their original institutional review board approvals and the Declaration of Helsinki. No new ethical approval was required for this synthesis of published literature.

## Conflicts of Interest

The authors declare no conflicts of interest.

## Data Availability

Data sharing not applicable to this article as no datasets were generated or analysed during the current study.
